# Clinical Approach to Pulp Canal Obliteration: A Case Series 

**DOI:** 10.22037/iej.v12i4.18006

**Published:** 2017

**Authors:** Kenia Maria Soares de Toubes, Patrícia Alves Drummond de Oliveira, Stephanie Nicácio Machado, Vânia Pelosi, Eduardo Nunes, Frank Ferreira Silveira

**Affiliations:** a *Itaúna University, Itaúna, MG, Brazil; *; b *Department of Pediatric Dentistry, FEAD, Belo Horizonte, MG, Brazil; *; c *Pontificial Catholic University of Minas Gerais, Belo Horizonte, MG, Brazil*

**Keywords:** Cone-beam Computed Tomography, Dental Operating Microscope, Digital Radiography, Guided Endodontics, Pulp Canal Obliteration, Ultrasound

## Abstract

This article describes four cases with safe and feasible clinical treatment strategies for anterior teeth with pulp canal obliteration (PCO) using cone-beam computed tomography (CBCT), digital radiography (DR), dental operating microscopy (DOM) and ultrasonic tips (US). Four anterior teeth with PCO were chosen. DR was taken with different angulations and analyzed with different filters. Subsequently, the access cavity was performed with the aid of DOM. If the canal was not identified, CBCT was requested. Sagittal and axial slices guided the direction of the ultrasonic tips. After identification of the canal, it was then negotiated and instrumented with the rotary instruments. All four canals were successfully identified, with no complications. In case 1, the canal was identified using DR, DOM and US tips. In cases 2, 3 and 4, the canals were identified with DR, DOM, US tips and CBCT. Complete root canal obliteration identified in radiography did not necessarily mean that pulp tissue was not visible clinically, either. The clinical evaluation of the access cavity with the aid of MO was crucial. If the canal was not identified, CBCT was mandatory in order to show more detailed view of the precise position of the canals, their directions, degrees of obstruction and dimensions. It served as a guide for the direction of the ultrasonic tips to keep them within the pulp chamber safely, with a low risk of iatrogenic injury.

## Introduction

Calcific metamorphosis, or pulp canal obliteration (PCO), is the pulp response to trauma, characterized by rapid deposition of mineralized tissue in the root canal space. Different factors, such as dental trauma, carious lesions, abfraction, abrasion, pulp capping, occlusal imbalance, orthodontic treatment, harmful oral habits and individual aging, can trigger PCO, which is becoming increasingly common [1, 2]. Generally, PCO has no symptoms and may be noted *via* tooth discoloration or routine examination [3-10]. There is controversy regarding whether endodontic treatment is indicated for teeth with PCO. Some authors recommend treatment only after appearance of symptoms and radiography shows apical bone rarefaction. However, others believe that immediate endodontic treatment is indicated because PCO may develop into an infection [5, 6, 11-14]. Digital radiographs of good quality, with the possibility of expansion and the use of different contrasts, are important to initiate the identification process. However, complete obliteration seen radiographically is not necessarily indicative of pulp tissue not being visible clinically or histologically [8, 15, 16]. The combination of dental operating microscopy (DOM) and ultrasonic tips (US) may help in identifying obliterated canals. DOM offers magnification and lighting, while ultrasonic tips allow working at greater depth within the pulp chamber safely, with a low risk of iatrogenic injury. However, in some situations, despite all of these resources and the skills and expertise of the operator, cone-beam computed tomography (CBCT) is necessary and allows three-dimensional images without overlapping adjacent structures, which facilitates the identification of the canals, their directions, degrees of obstruction and dimensions [8, 16-20]. Due to difficulty in managing these canals, proper diagnosis and careful planning before initiating endodontic treatment are needed.

**Figure 1:**
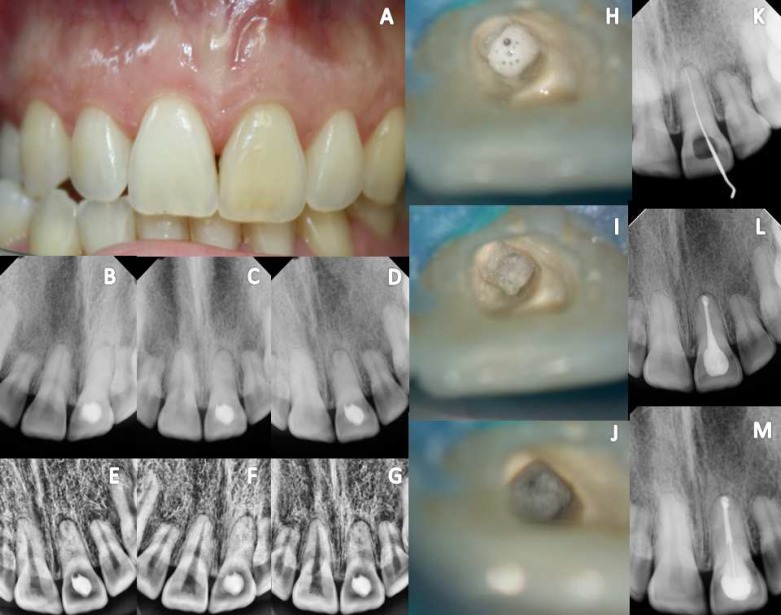
*Case 1* ***:*** A) *Maxillary left central incisor (21) showing marked discoloration; *B, C, D)* Digital periapical radiographs in the disto, ortho and mesio* *angulations; *E, F, G)* Evaluation of DR using the periodontics filter digital system XDR®; *H)* The access cavity was rectified and irrigated thoroughly with 5.25% sodium hypochlorite (NaOCl); *I)* A yellowish area was identified in the center of the tooth; *J)* A small hole was identified; *K)* DR - canal identified with K #10 file; *L)* DR-end fillings; *M)* Digital follow-up radiograph taken after 1 year showing complete healing of the lesion;* *no symptoms were present*

Currently, the introduction of new technologies has increased the predictability of these treatments and, consequently, their success rates [16, 20-22]. However, there is still little information in the literature to guide clinicians on how to use this new technology safely and effectively. Thus, this study aims to contribute to the knowledge of the clinical approaches used for anterior teeth with obliterated canals (PCO) by describing four clinical cases.

## Case Report

***Case 1:*** A 34-year-old woman presented with a history of trauma involving tooth 21 at 10 years of age. She underwent a three-year orthodontic treatment from 17 years of age. She initially presented to a general dentist due to darkening of the crown and the presence of swelling in the apical region. The dentist performed four unsuccessful canal identification attempts, and then the patient was referred to a specialist. Initially, clinical photographs were performed (Figure 1A). Digital radiographs (DR)(Digital system XDR®, São Paulo, Brazil) were performed in the ortho, distal and mesial angulations (Figure 1B, 1C, 1D). Through DR, a fine radiolucent line was identified at the center of the root, using a periodontic filter (Figure 1E, 1F, 1G). The patient was anesthetized, and a rubber dam was placed on the adjacent tooth. Then, the existing dressing was removed, and the access cavity was identified and irrigated thoroughly with 5.25% sodium hypochlorite (NaOCl) (Lenza Pharma, MG, Brazil) (Figure 1H). With the aid of DOM (DF Vasconcelos, São Paulo, SP, Brazil), a yellowish area was identified in the center of the tooth (Figure 1I). This area was thoroughly removed with ultrasonic tip (Helse, São Paulo, Brazil) coupled to an ultrasound machine ENAC (Osada, Inc.,California, EUA) set at low power.

A small orifice was identified (Figure 1J). AK C Pilotfile #10 (21.0 mm) (VDW®, Munich, Germany) was introduced with winding watch movements, with minimal vertical pressure, until the total root length was accessed (Figure 1K). Digital radiographies were taken in different angulations to confirm the correct position and the processes of cleaning and shaping were initiated. Endodontic therapy consisted of rotary instrumentation with ProTaper NEXT® (Dentsply Maillefer, was filled by vertical compaction (Figure 1L). A one-year follow-up radiography showed complete healing and the patient was asymptomatic (Figure 1M).

**Figure 2.**
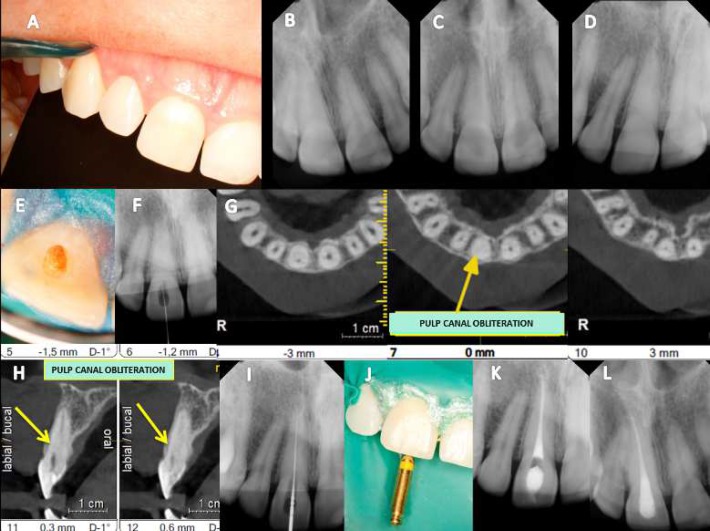
Case 2**:** *A)* Maxillary left central incisor (21) showing marked discoloration; *B, C, D)* Digital periapical radiographs in the disto, ortho and mesio angulations; *E, F, G)* Evaluation of DR using the periodontics filter digital system XDR®; *H)* The access cavity was rectified and irrigated thoroughly with 5.25% sodium hypochlorite (NaOCl); *I)* A yellowish area was identified in the center of the tooth; *J)* A small hole was identified; *K)* DR-canal identified with K #10 file; *L)* DR-end fillings; *M)* Digital follow-up radiograph taken after 1 year showing complete healing of the lesion. No symptoms were present

***Case 2:*** A 29-year-old woman was referred for endodontic treatment of the maxillary right central incisor due to a color change (Figure 2A). She reported no history of dental trauma. No clinical symptoms were reported, either and there was no evidence of occlusal trauma or a periodontal pocket. Digital radiographies were taken at multiple angles (Figure 2B to D). The pulp chamber was visible only in the middle and apical thirds. The patient was anesthetized, and a rubber dam was placed at a specified distance from the tooth that was to be treated. Pulp chamber cavity access was achieved with 3-4 mm of penetration parallel to long axis of the tooth with the aid of DOM (DF Vasconcelos, São Paulo, Brazil). At the entrance to the pulp chamber, dentin obstruction was observed at the cemento-enamel junction (CEJ).It was yellowish in color, which was suggestive of the original canal (Figure 2E). The pulp chamber was then flooded with 5.25% NaOCl (Lenza Pharma, MG, Brazil), and pulp tissue oxygenation bubbles were observed. Ultrasonic tips (Helse, São Paulo, Brazil) coupled to an ultrasound device ENAC were used to avoid excessive dentin removal. As the canal was not identified (Figure 2F), the cavity was filled with a small ball of sterile cotton and sealed with Coltosol cement (Coltene Whaledent, New York, USA) and flowable filling material Surefil (Dentsply Caulk, Derbyshire, England). CBCT was requested, and images were obtained in the axial and sagittal views and thoroughly evaluated. In the axial sections, the visible pulp chamber was identified only in the apical third (Figure 2G). In the sagittal sections, it was observed that erosion began when the ultrasonic tips were directed more buccally (Figure 2H). A second procedure was scheduled, and the cavity was re-opened. Using DOM, small burnouts directed toward the palatal were conducted with US tips. A small slot was identified as the canal *via* DOM (Figure 2I). Mechanical chemical preparation and obturation were performed as in the previous case (Figure 2J and 2K). Digital follow-up radiographs taken after 1 year showed complete healing and no symptoms were present (Figure 2L).

**Figure 3:**
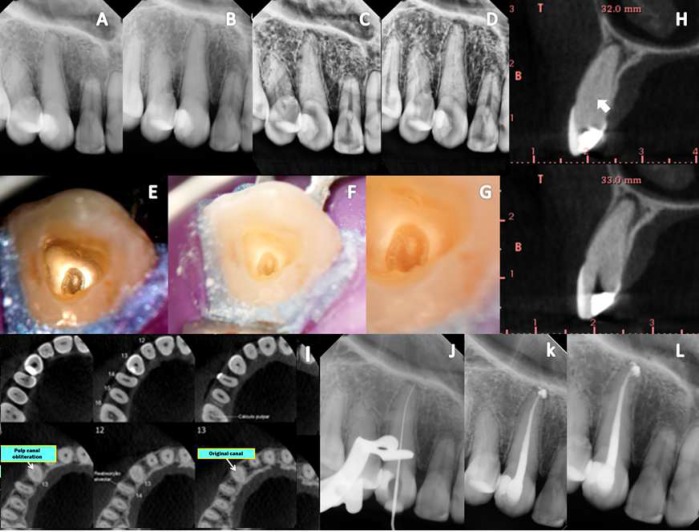
*Case 3: *A, B)* DR was performed at multiple angles before the cavity was accessed; *C, D)* Evaluation of DR using the periodontics filter digital system XDR®; *E, F, G)* Identification of a yellowish area, which was suggestive of the original canal, via DOM; *H, I)* CBCT sagittal and axial slices: obliteration of the canal in the cervical third and the presence of light in the middle and apical thirds (ARROWS); *J)* DR: canal identification; *K)* Final DR: canal fillings; *L)* Follow-up imaging after 1 year of treatment*

***Case 3:*** A 25 year-old woman presented with apical swelling and acute persistent pain involving tooth #13. The patient stated that the tooth had been orthodontically tractioned. Radiographic examination with different angulations and contrast showed thickening of the periodontal ligament and obliteration of the canal (Figure 3A to 3D). Initially, the patient was anesthetized, and a rubber dam was placed at a specified distance from the tooth. Access to the pulp chamber was achieved with the aid of DOM (DF Vasconcelos, SP, Brazil). The canal orifice was not identified (Figure 3E to 3G). Then, CBCT was requested. After evaluation *via* axial and sagittal imaging (Figure 3H and 3I) showing the real position of the canal, treatment was conducted similar to the previous cases (Figure 3J and 3K). A follow-up radiography taken 1 year after treatment showed complete healing, and no symptoms were reported (Figure 3L).

**Figure 4:**
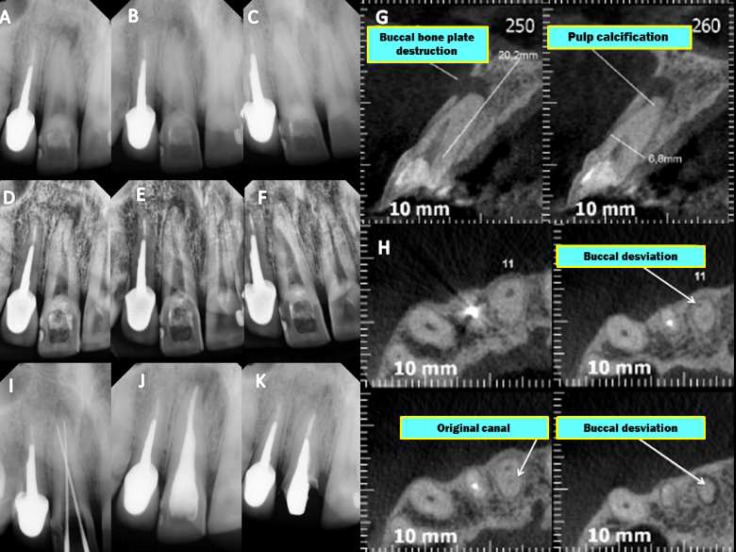
Case 4 *A, B, C)* Initial DR at different angulations; *D, E, F)* Evaluation of DR using the periodontics filter digital system XDR®; *G,H)* CTCB: sagittal and axial slices showing canal deviation identified buccally (ARROWS); *I)* DR with the identification of the original canal; *J)* Final radiograph after obturation; *K)* Follow-up radiograph taken 2 years after treatment

***Case 4:*** A 58 year-old man was referred for re-treatment of tooth #21. According to the patient, this tooth caused acute pain several years earlier but was not endodontically treated because the canal was not identified. The clinician opted to perform apical curettage. Clinically, the tooth had been stable for four years. However, the patient’s symptoms returned. The same clinician attempted to locate the canal using a probe file and EDTA, resulting in an apical perforation of the labial root surface. Then, the patient was referred to an endodontist. Initially, radiographs were taken (Figure 4A to F). Subsequently, CBCT was performed to analyze the findings (Figure 4G and H). The patient was anesthetized, and a rubber dam was placed on a specified distance from the tooth. With the aid of DOM and the axial and sagittal data gathered *via* CBCT, the perforation was identified (Figure 4I) and sealed with mineral trioxide aggregate (MTA Fillapex, Angelus, Londrina, Brazil). The true position of the canal was identified, and cleaning, shaping and obturation were conducted the same way as in case 1. A postoperative radiography was taken showing successful treatment (Figure 4J). A follow-up radiograph taken after one year showed complete healing of the lesion (Figure 4K). The patient was asymptomatic.

## Discussion

The current literature presents some articles describing the causes of pulp obliteration, but there still exists a paucity of protocols for the efficacious and safe treatment of PCO. Studies have shown that the success of endodontic therapy is based on the correct debridement, disinfection and obturation of the root canal system (RCS) [23]. Pulp obliterations can prevent access to the entrances of the canals, modify internal anatomy and divert inserted instruments [16, 24, 25]. Generally, the process of pulp obliteration confers an unfavorable prognosis because atypical morphology creates major challenges for treatment that increase the risk of iatrogenic complications [13, 26], making the outcome even more uncertain in cases of pulp necrosis [10, 24, 27-29]. 

Higher prevalence of obliterations occur in the central and lateral incisors [9, 26]. Several adjunctive utilities and techniques, such as ultrasound, chelating agents, magnification with indirect optical fibers, lateral radiographs, EDTA combined with NaOCl and endodontic explorers, are used for the implementation of endodontic therapy [26]. Chelating agents may be useful for locating obliterated canals not identified *via* other means [8]. However, the use of chelating agents (CA) and probe files is controversial in the literature, which corroborates the findings of our report of 4 anterior teeth with CPO that were successfully managed using DR, US tips, DOM and CBCT. CA absorb calcium ions, increasing the porosity of the dentin, which may induce the creation of false canal deviations and perforations, as presented in case 4 (Figure 4G and 4H). Therefore, the use of a probe file and SQ are inadvisable in the initial management of the canal.

The introduction of digital radiography (DR) in routine clinical practice in endodontics provides speed and agility in obtaining radiographic images, as well as expansion. It also allows the use of contrast and filters, which increase the possibility of identifying the canal (Figure 1E to 1G). DR should be performed at different angles, both initially and during treatment, to assess the depth and direction of the ultrasonic tips [30, 31].

When searching for hidden canals, it is crucial to note the level of the cemento-enamel junction (CEJ), which is the most consistent noticeable milestone denoting the location of the pulp chamber [32]. Usually, a rubber dam is placed with a butterfly clamp *in situ* [8]; however, this should be performed away from the tooth being treated because the clip prevents the display of the CEJ, and its removal allows recontamination of the SCR. Routine access in anterior teeth is achieved in the exact center of the palatal surface of the buccolingual and cervical section crown [4, 26] at a 45^°^ angle to the long axis of the tooth to reach an empty space, which may not occur in the case of CPO and may lead to a large number of iatrogenic complications. However, based on the axial and sagittal views obtained *via* CBCT, the canals are well centered in the tooth (Figure 2H, 3I and 4H) [4, 32], which allows access, and the cavity is prepared near or through the incisal edge [4, 7], avoiding the possibility of a perforation in the labial root surface and unnecessary damage. Generally, secondary dentine has a more whitened appearance, while obliterated pulp presents a darker color (Figure 3E to G). DOM is essential for the removal of calcified areas in the pulp chamber and within the deep canal [33, 34]. US tips, in contrast to drills, provide a more conservative approach to conventional treatment [25, 35]. US tips do not rotate inside the canal, ensuring greater security and control while maintaining their cutting efficiency [36]. They are useful for refining surgical access and help in breaking up calcifications covering the canal opening, which allows safe access to deeper areas, with enough safety and minimal wear, and the identification of dental structures [33].

In some situations, the identification of the canal *via* DR does not imply that the canal will be located clinically because its entrance may be blocked (Figure 2E and F). DR provides two-dimensional images of three-dimensional structures [31]. However, this deficit can be reduced with the aid of CBCT, which allows 3D displays without overlapping adjacent structures and visualizes the locations of the canals, their directions, degree of obstruction, dimensions and other important information [16-20, 37]. However, CBCT should not always be requested initially in cases of PCO. As demonstrated in this report, there was no need for CBCT in case 1. However, CBCT was of great value in cases 2, 3 and 4, in which the canal was unable to be identified by DR, DOM and US tips. Based on the data gathered from axial and sagittal slices, it was possible to identify the correct position of the canal, which showed the usefulness of CBCT as it served to guide the US tips in the correct direction, avoiding iatrogenic injury and minimizing costs and patient exposure to radiation, resulting in a favorable prognosis.

Guided endodontics seems to be a safe and clinically viable method of locating PCO [21, 22]. However, for the manufacture of the guided model, there is a need for high-tech equipment, such as scan-scanning intra oral 3D printer and the construction of a printed model, which results in higher cost for the patient. The diameters of the drills are still inadequate for lower teeth and fine roots [22]. In addition, the need to fix and stabilize the printed template that will guide a bur to the calcified root canal can create a bit of fear for less experienced clinicians in surgical procedures [38, 39].

## Conclusion

It is important to emphasize that the negotiation of small obliterated spaces is a tremendous challenge for clinicians. The use of new technologies, in addition to sufficient knowledge of pulp anatomy and radiographic techniques and patience, is the key to success in solving cases of PCO.
